# Regulation of the H1 Type VI Secretion System by the Transcriptional Regulator NfxB in *Pseudomonas aeruginosa*

**DOI:** 10.3390/ijms26041472

**Published:** 2025-02-10

**Authors:** Shuhui Liu, Ziyuan Wu, Wenbo Yan, Qian Liu, Yuanli Zhao, Tingting Gao, Yiming Yang, Linke Cao, Ruixue Tao, Meng Li, Lijun Liu, Yani Zhang, Tietao Wang

**Affiliations:** Key Laboratory of Resource Biology and Biotechnology in Western China, Ministry of Education, Provincial Key Laboratory of Biotechnology, College of Life Sciences, Northwest University, Xi’an 710069, China

**Keywords:** *Pseudomonas aeruginosa*, T6SS, NfxB, antibiotic resistance, bacterial competition

## Abstract

The type VI secretion system (T6SS) is a widely distributed molecular apparatus found in most Gram-negative bacteria. Studies show that T6SSs have functions in bacterial virulence, inter- and intra-bacterial competition, and environmental adaptation. *Pseudomonas aeruginosa*, an opportunistic pathogen, harbors three T6SS gene clusters that perform diverse roles in clinical infection. Herein, using DNA affinity chromatography of the H1-T6SS promoter, the fluoroquinolone antibiotic resistance regulator NfxB was identified. Further studies demonstrated that NfxB negatively regulates the expression of H1-T6SS by directly binding to its promoter region. T6SS expression and effector secretion are regulated by the fluoroquinolone antibiotic via NfxB, which enhances inter-bacterial competition in the complex bacterial ecology. Meanwhile, the deletion of *nfxB* alters carbenicillin resistance through an unknown pathway. This study provides new insights into the regulation of T6SS by environmental signals, and it provides data support for antibiotic resistance and inter-bacterial competition due to T6SSs.

## 1. Introduction

The type VI secretion system (T6SS) is a complex puncturing apparatus that is widely distributed in more than 25% of sequenced Gram-negative bacteria genomes [1]. In many bacteria, genomes harbor multiple T6SS clusters that contribute to bacterial virulence, interbacterial competition, and environmental adaptation [2,3]. The T6SS contains 14 key conserved structural proteins and several accessories or secreted proteins, which form a fully functional “molecular syringe” [4]. *P. aeruginosa* is an opportunistic human pathogen that is wildly distributed in the environment [5]. *P. aeruginosa* encodes a variety of toxin-related effectors, such as cytotoxicity, immune evasion and motility abilities, antibiotic resistance, and biofilm structure, and it regulates bacterial infection and virulence [6]. Bacterial secretion systems play an important role in bacterial pathogenicity and competition. In *P. aeruginosa*, three T6SS loci have been characterized, and they are named H1-, H2-, and H3-T6SS; they are involved in a variety of physiological functions [7,8]. Due to their structural complexity and diverse functions, the expression of T6SSs is strictly regulated by relevant environmental signals. In *P. aeruginosa*, quorum sensing (QS) and RetS-Gac-Rsm systems are two major global regulatory pathways of T6SSs that regulate bacterial pathogenesis and competitive abilities [9,10].

Multiple antibiotic resistance is an important feature of *P. aeruginosa*, and it is controlled by changes in membrane permeability, efflux pumping systems, antibiotic-inactivating enzyme acquisition, and biofilm formation [11]. Antibiotic resistance is regulated primarily through horizontal gene transfer, spontaneous mutations at the genome level, and gene expression regulation. NfxB is a TetR/AcrR family transcriptional regulator for the *mexCD-oprJ* operon and fluoroquinolone antibiotic resistance [12,13]. Previous studies have shown that mutations in *nfxB* enhance antimicrobial resistance by overexpressing the multidrug efflux pump by directly derepressing the *mexCD-oprJ* promoter [14]. Recent research reveals that altering the efflux pump affects virulence factor (elastase, protease IV, pyocyanin, and rhamnolipids) production and swarming ability inhibition via defects in the quorum sensing (QS) system [15].

Previous studies have revealed that NfxB functions in multidrug resistance and bacterial virulence [16]. Herein, our promoter affinity assay revealed that NfxB negatively regulates the expression of H1-T6SS by directly binding its promoter region. Further studies found that NfxB regulates Tse1 secretion, a key antibacterial effector of T6SS, in response to fluoroquinolone antibiotics, thereby enhancing inter-bacterial competition in complex bacterial ecosystems. The findings revealed a novel cross-regulation model between antibiotics and secretion systems in environmental pathogens.

## 2. Results

### 2.1. NfxB Negatively Regulates the Expression of H1-T6SS

**Figure 1:**
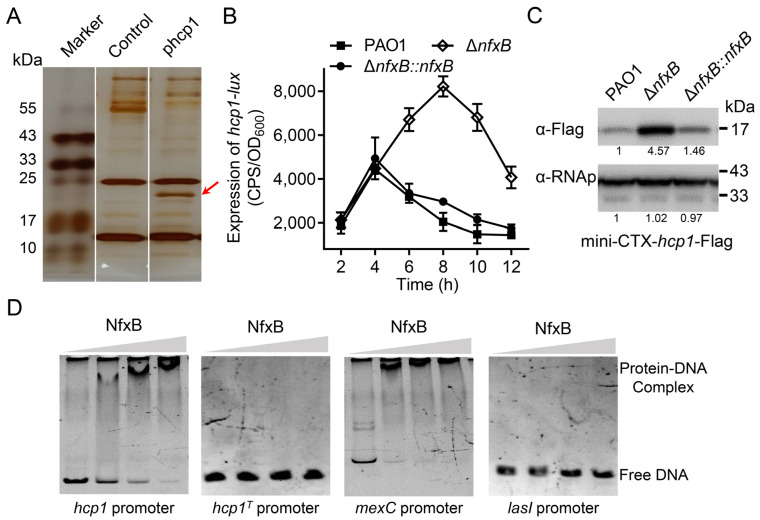
NfxB negatively regulates the expression of H1-T6SS in *P. aeruginosa*. (**A**) Identification of NfxB as the H1-T6SS *hcp1* promoter binding protein. Biotin-labeled *hcp1* promoter (phcp1) or *hcp1* open reading frame (control) DNA fragments coated with Dynabeads MyOne Streptavidin C1 beads were incubated with PAO1 cell lysates. After sufficient washing with PBS, the proteins were resolved using SDS-PAGE electrophoresis and visualized using silver staining. The bands retained by the *hcp1* promoter but not control DNA were excised and analyzed with LC-MS/MS. The protein band for mass spectrometry is marked with a red arrow. (**B**) The promoter activity of *hcp1* was measured in the wild-type PAO1, Δ*nfxB* mutant, and complementary strains. The indicated bacteria were cultured in LB medium at 37 °C for 12 h. The expression of *hcp1*-lux and the optical density (600 nm) of each sample were obtained at the indicated time. (**C**) A mini-CTX plasmid directing the expression of the Hcp1-Flag chimera driven by the native promoter was integrated into derivative strains *P. aeruginosa*. The protein level of Hcp1-Flag from the indicated strains was examined with a Western blot. The RNA polymerase α (RNAp) antibody was used as a loading control. The intensity of bolt bands was quantified using Fiji ImageJ (version 1.54i). (**D**) NfxB binds to the promoter region of *hcp1*. Promoter fragments of *hcp1*, *hcp1* truncated (*hcp1^T^*), *mexC*, or *lasI* promoter were incubated with 0, 0.05, 0.1, or 0.15 μM purified NfxB protein in Tris buffer at room temperature for 20 min. Then, the reaction mixtures were analyzed using PAGE electrophoresis and visualized using SYBR gold dye (TransGen Biotech, Beijing, China). The *mexC* promoter was a positive control. The *lasI* promoter was a negative control. (**A**,**C**,**D**) Similar results were obtained from three independent experiments, and the images shown are from one representative experiment. (**B**) The data shown are the average of three independent experiments; error bars indicate the SD from three independent experiments.

H1-T6SS is an important secretion apparatus in *P. aeruginosa*, and it is involved in bacterial competition and pathogenesis [2,17]. To further investigate the regulatory mechanisms of H1-T6SS, a DNA pull-down assay was carried out with streptavidin-coupled magnetic beads and the key promoter of the H1-T6SS gene cluster. After affinity chromatography of *P. aeruginosa* cell lysate using *hcp1* promoter DNA, a significant protein band was observed in the SDS-PAGE gel (Figure 1A). Based on mass spectrometry, the protein was identified as NfxB (Figure S1), a TetR/AcrR family transcriptional regulator for the *mexCD-oprJ* operon and fluoroquinolone antibiotic resistance [12,13]. To verify the regulatory relationship of NfxB and H1-T6SS, an in-frame deletion mutant strain Δ*nfxB* was constructed. Then, *hcp1*-promoter-driven *luxCDABE* reporter plasmid pKD-*hcp1* was transformed into wild-type PAO1 and the Δ*nfxB* mutant. As shown in Figure 1B, the luminescence intensity of the *hcp1* promoter–reporter fusion in the Δ*nfxB* mutant strain was significantly higher than that in the wild-type PAO1 strain, and the luminescence intensity could be restored to the wild-type level through *nfxB* complementation (Figure 1B). This regulation was also confirmed by monitoring the protein production levels of Hcp1 (Hcp1-Flag) with a Western blot assay in the total cell lysates of the indicated *P. aeruginosa* strains. In line with the *hcp1* gene transcription, the protein level of Hcp1 was much higher in the Δ*nfxB* mutant than in the wild-type strain (Figure 1C). To further characterize the gene expression of the H1-T6SS gene cluster regulated by NfxB, the three predicted promoters, *pfha1*, *ptssA1*, and *ptagJ1*, of H1-T6SS were fused with the *lux* reporter and transformed into the wild-type PAO1 or Δ*nfxB* mutant. The expression of the promoter was significantly higher in the Δ*nfxB* mutant than in PAO1 (Figure S2). These data suggest that NfxB negatively regulates the expression of H1-T6SS in *P. aeruginosa*.

### 2.2. NfxB Directly Binds to the hcp1 Promoter

NfxB is a TetR/AcrR family transcriptional regulator that represses fluoroquinolone antibiotic resistance by directly binding to the promoter region of the *mexCD-oprJ* operon [12,14]. NfxB is a potential binding regulator of the *hcp1* promoter, as indicated by DNA affinity chromatography, which implies that NfxB is a direct regulator of H1-T6SS. To test whether NfxB controls *hcp1* expression directly, we purified His_6_-NfxB using *E. Coli* BL21 (DE3/pET28a-*nfxB*) and amplified the promoter region of *hcp1* using PCR. Then, an electrophoretic mobility shift assay (EMSA) was performed using the *hcp1* promoter and purified His_6_-NfxB. Similarly to the known positive control *mexC* promoter, protein-DNA complexes were formed in NfxB and *hcp1* promoter DNA (Figure 1D). To further analyze the binding motif of NfxB, the sequence of the hcp1 promoter was analyzed with the known NfxB binding motif [14]. The *hcp1* promoter truncated the potential binding motif sequence (G**G**C**T**G**TTGAT**ATGTTC**ATCAA**G**A**T**C**G), which was analogous to the *mexC* promoter, and it lost the combination capacity for NfxB (Figure 1D). Thus, the above data demonstrate that NfxB directly binds to the *hcp1* promoter of H1-T6SS and represses H1-T6SS expression.

### 2.3. Deletion of nfxB Leads to Antibiotic Resistance

**Figure 2:**
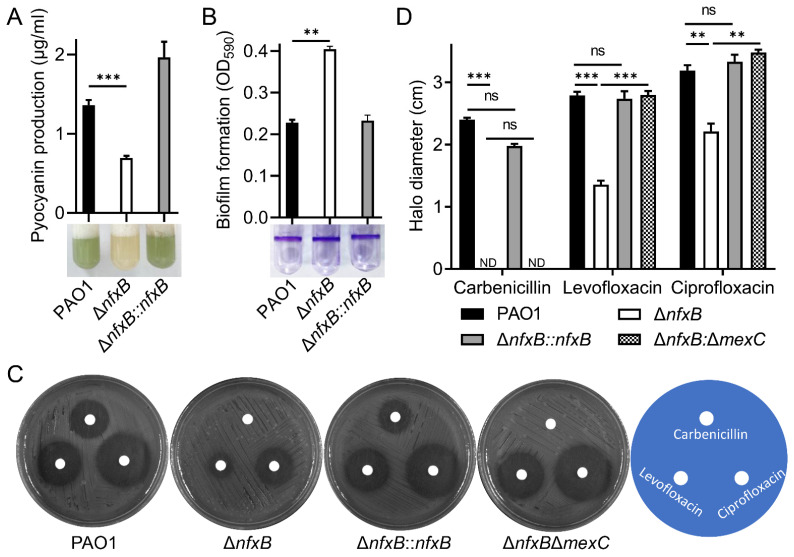
Deletion of *nfxB* leads to antibiotic resistance. (**A**,**B**) NfxB controls pyocyanin production and biofilm formation in *P. aeruginosa*. The pyocyanin production (**A**) or biofilm formation (**B**) of the wild-type PAO1, Δ*nfxB* mutant, and complementary strains was determined after culturing in LB medium at 37 °C for 12 h. (**C**,**D**) NfxB regulates the antibiotic resistance and susceptibility of *P. aeruginosa* PAO1. Cultures of the indicated wild-type PAO1, Δ*nfxB* mutant, complementary, and Δ*nfxB*Δ*mexC* mutant strains were plated on LB solid medium. Then, 10 μL of carbenicillin (10 mg/mL), levofloxacin (0.5 ug/mL), or ciprofloxacin (0.5 mg/mL) was spotted on a paper disk. Plates were incubated at 37 °C for 12 h; photographs of the clearing-resistant zone are shown (**C**), and inhibition halo diameter was measured (**D**). (**A**,**B**,**D**) The data shown are the average of three independent experiments; error bars indicate the SD from three independent experiments. Statistical significance was calculated using one-way ANOVA and Dunnett’s multiple comparison test; ** *p* < 0.01; *** *p* < 0.001; n.s., not significant. (**C**) Similar results were obtained from three independent experiments, and the images shown are from one representative experiment.

NfxB is a transcriptional regulator in metabolic changes and fluoroquinolone antibiotic resistance [12,15,16]. Consistent with a previous report, mutations in *nfxB* lead to pyocyanin production inhibition and increased biofilm formation (Figure 2A,B). Previous studies have shown that mutations in *nfxB* increase fluoroquinolone antibiotic resistance [13]. In this study, we found that the Δ*nfxB* mutant exhibited a strong carbenicillin resistance phenotype compared with the wild-type PAO1 during the construction of the complemented strain of the Δ*nfxB* mutant using a carbenicillin resistance plasmid. To further verify the superior carbenicillin resistance of the Δ*nfxB* mutant, we tested the carbenicillin inhibition zone using a paper disk assay. As shown in Figure S3, treatment with 10 mg/mL carbenicillin resulted in a clear inhibition zone for wild-type PAO1, but no inhibition zone was observed for Δ*nfxB*. More precisely, the minimum inhibitory concentration (MIC) of carbenicillin for the wild-type PAO1 was 150 ug/mL, and that for Δ*nfxB* exceeded 5000 ug/mL. In addition, the heightened carbenicillin resistance phenotype could be restored to the wild-type level through *nfxB* complementation with a chromosome integrating a mini-CTX-*nfxB* plasmid. The data suggest that mutations in *nfxB* lead to super carbenicillin resistance in *P. aeruginosa*.

The fluoroquinolone antibiotic resistance mediated by NfxB depends on the MexCD-OprJ efflux pump [15]. The MexCD-OprJ multidrug resistance efflux pump is composed of the cytoplasmic membrane fusion protein MexC, the membrane-spanning protein MexD, and the outer membrane porin protein OprJ, which function in antimicrobial resistance and micro-molecule transport [18,19]. To further explore the carbenicillin resistance characteristics of NfxB, the double mutant Δ*nfxB*Δ*mexC* was constructed, and susceptibility to carbenicillin, levofloxacin, or ciprofloxacin antibiotics was detected using a paper disk assay. According to previous studies, the deletion of *nfxB* results in levofloxacin and ciprofloxacin sensitivity, and the sensitivity was restored to the level of the wild-type PAO1 through the double deletion of the *mexC* gene, the key component of the multidrug efflux pump MexCD-OprJ, in the Δ*nfxB* mutant strain (Figure 2C,D). To test whether the superior carbenicillin resistance of Δ*nfxB* depended on the MexC component of the efflux pump, the inhibition zones for PAO1, Δ*nfxB*, Δ*nfxB::nfxB*, and Δ*nfxB*Δ*mexC* were examined using a paper disk assay. As shown in Figure 2C,D, unlike the *nfxB* complementation strain Δ*nfxB::nfxB*, the double-mutant strain Δ*nfxB*Δ*mexC* still exhibited heightened carbenicillin resistance (Figure 2C,D). More precisely, the double-mutant strain Δ*nfxB*Δ*mexC* exceeded 5000 µg/mL, similar to the single-mutant Δ*nfxB* strain. The data show that NfxB regulates fluoroquinolone antibiotic resistance via the multidrug efflux pump MexCD-OprJ, and the deletion of *nfxB* confers heightened carbenicillin resistance.

### 2.4. H1-T6SS Is Responsive to Fluoroquinolone Antibiotics in an NfxB-Dependent Manner

NfxB is a TetR/AcrR family transcriptional regulator for the *mexCD-oprJ* operon, which targets fluoroquinolone antibiotic response and resistance in *P. aeruginosa* [12]. Antibiotic resistance provides a growth advantage for bacteria in diverse environments and during host infection [20]. H1-T6SS in *P. aeruginosa* plays an important role in inter- and intra-bacterial competition [21]. Herein, we found that NfxB negatively regulates H1-T6SS in *P. aeruginosa*. Therefore, we hypothesized that the expression of H1-T6SS is regulated by antibiotics via the transcriptional regulator NfxB.

**Figure 3:**
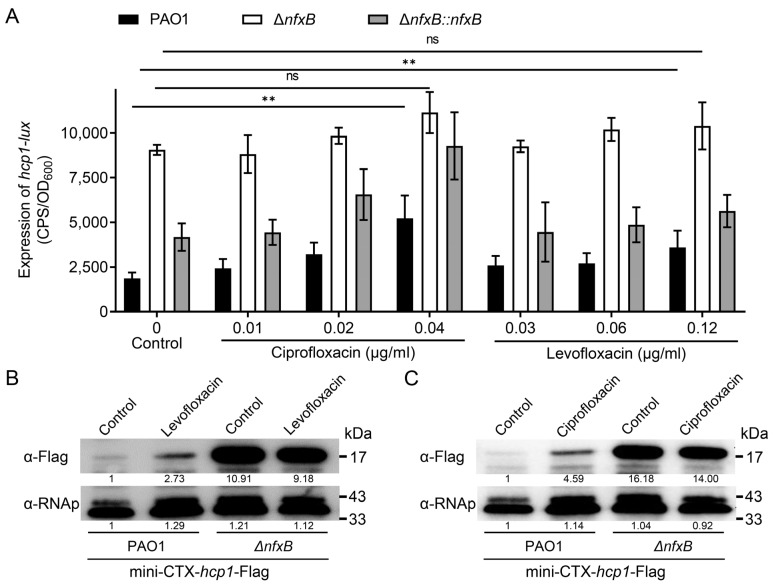
Expression of H1-T6SS is regulated by quinolone antibiotics via NfxB. The promoter activity of *hcp1-lux* (**A**) and Western blot (**B**,**C**) analysis of the expression of Hcp1 in *P. aeruginosa*. (**A**) Wild-type PAO1 and Δ*nfxB* mutant strains harboring a pKD-*hcp1-lux* in LB medium with the indicated concentrations of levofloxacin or ciprofloxacin at 37 °C for 8 h. (**B**,**C**) The native promoter driving Hcp1-Flag chimera was integrated into the wild-type PAO1 and Δ*nfxB*, and the bacteria were cultured in LB or LB containing 0.125 μg/mL levofloxacin (**B**) or 0.04 μg/mL ciprofloxacin (**C**) at 37 °C for 12 h. The protein level of Hcp1-Flag from the indicated strains was examined with a Western blot against the Flag antibody. The RNA polymerase α (RNAp) antibody was used as a loading control. The intensity of bolt bands was quantified using Fiji ImageJ. (**A**) The data shown are the average of three independent experiments; error bars indicate the SD from three independent experiments. Statistical significance was calculated using one-way ANOVA and Dunnett’s multiple comparison test, ** *p* < 0.01; n.s., not significant. (**B**,**C**) Similar results were obtained from three independent experiments, and the images shown are from one representative experiment.

To this end, two representative fluoroquinolone antibiotics, levofloxacin and ciprofloxacin, were selected to verify the regulation mode of NfxB. After culturing PAO1 and Δ*nfxB* mutant strains in LB medium for 12 h with a sub-inhibitory concentration of levofloxacin or ciprofloxacin, the luminescence intensity of the *hcp1*-promoter-driving *lux* reporter in the indicated strains was quantified using a Synergy 2 plate reader. As shown in Figure 3A, in the wild-type PAO1, the expression of *hcp1* was significantly upregulated at various concentrations of levofloxacin or ciprofloxacin (Figure 3A), but these changes were not observed in the Δ*nfxB* mutant strain (Figure 3A). To further verify the relationship between fluoroquinolone antibiotics and the H1-T6SS promoter, the Hcp1 (Hcp1-Flag) production level in the wild-type PAO1 and Δ*nfxB* mutant strains was determined with a Western blot using Flag antibody. Consistently with the *lux* luminescence reporter assay, the Hcp1 production level was approximately upregulated 2.73- or 4.59-fold by levofloxacin (Figure 3B) or ciprofloxacin (Figure 3C) in the wild-type PAO1, but not in the Δ*nfxB* mutant strain (Figure 3B,C). The results indicate that the expression of *hcp1* in the wild-type PAO1 was repressed by NfxB, and this repression was alleviated by levofloxacin or ciprofloxacin. Taken together, our results strongly demonstrate that H1-T6SS expression is responsive to fluoroquinolone antibiotics via NfxB.

### 2.5. NfxB Regulates T6SS-Dependent Bacterial Competition

**Figure 4:**
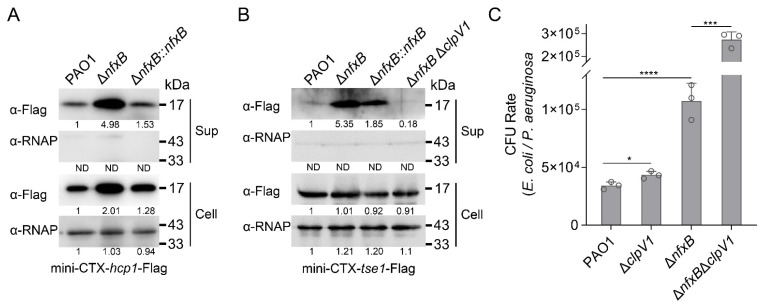
NfxB regulates H1-T6SS-dependent bacterial competition. (**A**) NfxB regulates the expression and secretion of Hcp1 in *P. aeruginosa*. The native promoter driving Hcp1-Flag chimera was integrated into the chromosomes of the wild-type PAO1, Δ*nfxB* mutant, and complementary strains. (**B**) NfxB regulates the secretion of Tse1 in *P. aeruginosa*. The native promoter driving Tse1-Flag chimera was integrated into the chromosomes of the wild-type PAO1, Δ*nfxB* mutant, Δ*nfxB*-complemented, and Δ*nfxB*Δ*clpV1* double-mutant strains. (**A**,**B**) The bacteria were cultured to OD_600_ = 1.0 in LB medium at 37 °C, and then the Flag-tagged proteins in the cell (Cell) and concentrated supernatant (Sup) protein fractions were examined using a Western blot against the Flag antibody. The RNA polymerase α (RNAp) antibody was used as a loading control. The intensity of bolt bands was quantified using Fiji ImageJ. (**C**) NfxB negatively controlled H1-T6SS’s mediation of interbacterial competition. An interbacterial killing assay between the indicated *P*. *aeruginosa* strains and *E*. *coli* was conducted on solid LB-LS medium at 37 °C for 12 h. The CFU rate was quantified after growth competition assays between the indicated organisms. (**A**,**B**) Similar results were obtained from three independent experiments, and the images shown are from one representative experiment. (**C**) The data shown are the average of three independent experiments; error bars indicate the SD from three independent experiments. Statistical significance was calculated using one-way ANOVA and Dunnett’s multiple comparison test; **** *p* < 0.0001; *** *p* < 0.001; * *p* < 0.05.

*P. aeruginosa* H1-T6SS secretes multiple effectors targeting bacterial and host cells, including Tse1, Tse2, and Tse3 [17,21]. The secretion of effectors is dependent on the integrated components of the T6SS, in which the *clpV* gene encodes an AAA+ family ATPase that provides energy to drive the secretory apparatus [7]. Our study revealed that the expression of H1-T6SS is responsive to fluoroquinolone antibiotics via the transcriptional regulator NfxB. To test whether the protein secretion of H1-T6SS is regulated by NfxB, mini-CTX-*hcp1*-Flag or mini-CTX-*tse1*-Flag production plasmids with Hcp1 and a representative effector, Tse1 were integrated into *P. aeruginosa* chromosomes, respectively. Then, the Hcp1 and Tse1 protein secretion and production were collected, and the protein levels were detected with a Western blot. The Hcp1 production and secretion were significantly upregulated in the Δ*nfxB* mutant strain, and the protein levels were restored to the wild-type level in the complementary strain (Figure 4A). Consistently with the regulation of the Hcp1 protein, the Tse1 production and secretion were negatively regulated by NfxB (Figure 4B).

H1-T6SS and its secreted effectors are engaged in bacterial competition for a growth advantage. To this end, an inter-bacterial competition assay was carried out between *P. aeruginosa* and *E. Coli* on solid plates in a contact-dependent manner. Unexpectedly, the Δ*nfxB* mutant strain did not exhibit significantly higher competitive activity than that of the wild-type PAO1 (Figure 4C). Previous studies have reported that high pyocyanin production enhances the bacterial competition of *P. aeruginosa* [22,23]. To further characterize the inter-bacterial competition mediated by H1-T6SS, a double-mutant strain Δ*nfxB*Δ*clpV1* was constructed, and an interbacterial competition assay was performed with *E. Coli* as prey. As shown in Figure 4C, the recovery of *E. Coli* was significantly higher with the Δ*nfxB*Δ*clpV1* mutant strain than with the Δ*nfxB* strain (Figure 4C). These data indicate that the secretion of H1-T6SS is involved in inter-bacterial competition and is regulated by NfxB in *P. aeruginosa*.

## 3. Discussion

T6SSs are an important molecular apparatus for bacterial virulence, inter-bacterial competition, and environmental adaptation. They are regulated by diverse transcriptional regulators and environmental signals. In *P. aeruginosa*, three T6SS clusters are regulated by diverse systems. The expression of H1-T6SS is controlled by the threonine phosphorylation system, PppA-PpkA, and the master regulatory pathways, RetS-Gac-Rsm [7,9,24,25]. Meanwhile, the quorum sensing (QS) system regulates the expression of three T6SSs differentially [10]. The expression of H1-T6SS is suppressed by the transcriptional regulators LasR and MvfR [10]. Here, we found that NfxB negatively regulates H1-T6SS by directly binding to the promoter region. The expression of H1-T6SS is regulated by fluoroquinolone antibiotics via NfxB to enhance the inter-bacterial competition in complex bacterial ecosystems.

NfxB is a negative regulator of the quinolone efflux system MexCD-OprJ, which responds to and plays an important role in environmental antibiotic resistance [12,13,14]. Through promoter affinity chromatography and mass spectrometry, we found that NfxB binds to the promoter of H1-T6SS and regulates the expression of H1-T6SS via fluoroquinolone antibiotics. A previous study showed that NfxB responds to antibiotic stress through its tetrameric formation, which is mediated by an antibiotic signal [14,26]. As the fluoroquinolone antibiotic concentration increases in a bacterial biotope, the cells sense the antibiotic signals and derepress the expression of *mexCD-oprJ* genes related to the quinolone antibiotic efflux pump, which enhances their antibiotic resistance. Meanwhile, the expression of H1-T6SS is derepressed, thereby promoting environmental adaptability and a competitive advantage in a complex ecological environment.

H1-T6SS is primarily involved in host cell toxicity and bacterial competition [27], and the expression of H1-T6SS is regulated by master transcriptional regulators, such as the RetS-Gac-Rsm and QS pathways. Our previous studies showed that, in *Yersinia pseudotuberculosis*, the expression of T6SS4 was found to be regulated by a variety of transcriptional regulators, such as OxyR and HpaR for oxidative stress response and Zur and ZntR for low zinc ion environments [28,29,30,31]. In *P. aeruginosa*, H2-T6SS is regulated by CueR and Anr, which respond to environmental copper concentrations and low oxygen [32,33]. Here, we found that the expression of H1-T6SS is regulated by an antibiotic-related regulator, NfxB; the results reveal a novel relationship among environmental adaptation, antibiotic resistance, and bacterial virulence, and they establish a connection between antibiotics and inter-bacterial competition.

**Figure 5:**
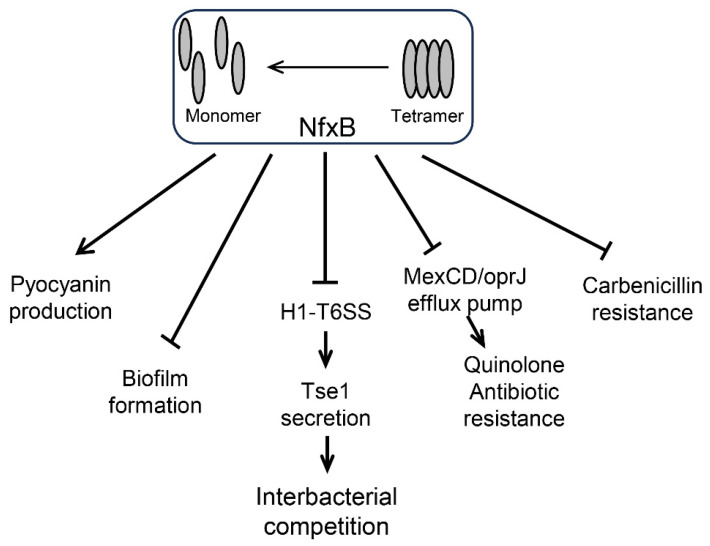
NfxB regulates pathways in *P*. *aeruginosa*. (i) NfxB positively regulates pyocyanin production by inhibiting the quorum sensing (QS) response via HHQ extrusion with the multidrug resistance efflux pump MexCD-OprJ [15,26]. (ii) Biofilm formation is negatively controlled by NfxB via an unknown pathway [36]. (iii) NfxB negatively regulates H1-T6SS expression and Tse1 secretion for interbacterial competition. (iv) NfxB negatively regulates quinolone antibiotic resistance via the MexCD/OprJ efflux pump [14]. (v) Carbenicillin resistance is negatively regulated by NfxB via an unknown pathway.

Antibiotic stress strongly promotes the intracellular formation of reactive oxygen species (ROS) [34]. These results are consistent with previous reports on *Y. pseudotuberculosis* and *Burkholderia thailandensis*, where T6SSs were found to be regulated by antioxidation-related transcription regulators [31,35]. This study reveals a new transcriptional regulator related to the environmental adaptation of T6SSs, which enriches the research on the expression regulation of bacterial secretion systems. NfxB is a long-known transcriptional regulator that is involved in various bacterial life processes. This work found that the deletion of *nfxB* significantly alters pyocyanin production and biofilm formation, which is consistent with previous reports [15,36]. Unexpectedly, the deletion of *nfxB* remarkably altered carbenicillin resistance in *P. aeruginosa* PAO1. A further study showed that the carbenicillin resistance ability does not depend on MexC, and how NfxB alters carbenicillin resistance and the relationship with MexCD-OprJ needs to be further studied. Overall, these results revealed a novel function of NfxB, which regulates the expression of T6SSs via antibiotics, extends the understanding of T6SS regulation, and provides insight into antibiotic resistance and inter-bacterial competition. Figure 5 reflects the key points of the Section 3 (Discussion).

## 4. Materials and Methods

### 4.1. Bacterial Strains and Growth Conditions

The bacterial strains and plasmids used in this study are listed in Table S1. *P*. *aeruginosa* PAO1 and its derivatives were cultured in Luria–Bertani medium (1% tryptone, 0.5% yeast extract, 1% NaCl) or *Pseudomonas* isolation agar (PIA) at 37 °C with appropriate antibiotics added to maintain the plasmid. *E. coli* strains were cultured in LB medium at 37 °C with appropriate antibiotics added to maintain the plasmid. The concentrations of the antibiotics are shown below; 50 μg/mL kanamycin, 10 μg/mL tetracycline, and 100 μg/mL carbenicillin were used for *E. coli*, and 100 μg/mL tetracycline,1200 μg/mL trimethoprim, and 300 μg/mL carbenicillin were used for *P. aeruginosa*.

### 4.2. DNA Pull-Down Assay

A DNA pull-down assay was carried out as previously described with minor modifications [37]. To analyze the binding proteins of the key promoter *hcp1* of H1-T6SS, a fragment of the *hcp1* promoter or open-reading-frame region was amplified using biotin-labeled primer pairs, as shown in Table S2. Then, 30 μL of PBS-equilibrated Dynabeads MyOne Streptavidin C1 (Thermo, Waltham, MA, USA) were co-incubated with the indicated DNA probes in PBS at 37 °C for 2 h. The uncoated DNA probes were then washed away with PBS and further incubated with PAO1 cell lysates for another 4 h. Finally, after sufficient washing with PBS, the proteins were resolved through SDS-PAGE electrophoresis and visualized using silver staining. The band retained by *hcp1* promoter DNA, but not by *orf* control, was excised and analyzed through LC-MS/MS.

### 4.3. LC-MS/MS Analysis

Mass spectrometry was carried out as previously described with minor modifications [33]. The excised protein band sample was subjected to in-gel trypsin digestion, and the tryptic peptides were extracted from the gel by equilibrating the sample with 50% acetonitrile and 5% formic acid. Then, the resulting peptide sample was vacuum-dried and reconstituted in HPLC-grade water. Ultimately, an Orbitrap Exploris 240 mass spectrometer(Thermo, Waltham, USA) combined with a Dionex UltiMate 3000 RSLCnano System (Thermo, Waltham, USA) was utilized for LC-MS/MS analyses, as previously described [38]. The MS/MS raw data were searched against the *P. aeruginosa* PAO1 protein database (downloaded from http://www.pseudomonas.com (accessed on 10 October 2021)) according to the manufacturer’s instructions.

### 4.4. Construction of Plasmids and Deletion Mutant Strains

The primers used in this study are listed in Table S2. The *nfxB* in-frame deletion mutant was constructed as described previously [33,39]. Briefly, 1060 bp of material up-stream and 1060 bp of material down-stream of the *nfxB* gene were amplified with PCR using pEX-*nfxB*-Up-S/A and pEX-*nfxB*-Down-S/A primer pairs. The two PCR products were digested and cloned into a similarly digested pEX18Tc plasmid to obtain pEX18Tc-*nfxB*. Then, the plasmids were electroporated into the wild-type PAO1, and the colonies were selected according to tetracycline susceptibility and sucrose (15%) resistance. The Δ*nfxB* mutant strains were verified using PCR and confirmed using DNA sequencing. For Δ*nfxB*Δ*mexC* double-mutant construction, 1539 bp of material up-stream and 1495 bp of material down-stream of the *mexC* gene were used to obtain the pEX18Ap-*mexC* plasmid, and Δ*nfxB*Δ*clpV1* double mutants were constructed using pEX18Tc-*nfxB* and the Δ*clpV1* mutant strain using a similar approach to that described in a previous study [33].

To detect the promoter expression activity of the *fha1*, *tssA1*, and *tagJ1* genes of H1-T6SS, the promoter region of the indicated gene was amplified with PCR using the corresponding primers. Then, the promoter fragment was digested with a restriction enzyme and cloned into a similarly digested pMS402 plasmid to yield pKD-*fha1*, pKD-*tssA1*, or pKD-*tagJ1*. To obtain NfxB expression plasmids, the fragment of the *nfxB* open reading frame was amplified with PCR using the corresponding primers. Then, the DNA fragments and pET28a plasmid were digested with the indicated restriction enzyme and cloned into pET28a to yield the pET28a-*nfxB* plasmid.

To construct a chromosome integration complementation plasmid, the promoters and the entire gene of *nfxB* were amplified with PCR using the corresponding primers. Then, the DNA fragments were cloned into a similarly digested mini-CTX-lacZ-Flag plasmid to yield a mini-CTX-lacZ-*nfxB* plasmid. The mini-CTX-*hcp1*-Flag, mini-CTX-*tse1*-Flag, mini-CTX-*nfxB*-*tse1*-Flag, and mini-CTX-*nfxB*-*hcp*1-Flag plasmids were constructed using a similar approach with the corresponding primers listed in Table S2.

### 4.5. lux-Based Gene Expression Assay

The expression of *lux*-based reporters was measured as previously described [40]. Overnight cultures of the indicated reporter strains were diluted at 1:100 with fresh LB medium and inoculated into parallel wells on a black 96-well plate with a transparent bottom. Then, the bacteria were further cultured at 37 °C for 12 h in a Synergy 2 plate reader (Biotek, Winooski, VT, USA). The luminescent light production (in counts per second) and optical density (600 nm) of each sample were obtained using the Synergy 2 plate reader (Biotek). For gene expression analysis, the luminescence intensity was normalized with the optical density and is shown as CPS/OD_600_.

### 4.6. Western Blot Assay

Overnight bacterial cultures were diluted to 1% in fresh LB medium and sub-cultured to OD_600_ = 2.0 at 37 °C. Then, 0.5 mL of cell culture was collected through centrifugation and dissolved in 50 μL of 1×SDS loading buffer. After boiling at 100 °C for 5 min, 5 μL of each soluble protein sample was separated using 15% SDS-PAGE and transferred onto a PVDF membrane (Millipore, Billerica, MA, USA). After blocking with 5% skimmed milk, the tagged proteins were hybridized with Flag or RNA polymerase α antibodies, respectively, and HRP-conjugated secondary antibodies to enhance the signals. Finally, the tagged proteins were visualized with an ECL Plus Kit (Tanon Technologies, Shanghai, China) using a Tanon 5200 Image analysis system (Tanon Technologies, Shanghai, China) according to the manufacturer’s instructions.

### 4.7. Expression and Purification of NfxB Protein

To express the His_6_-tagged NfxB protein, the pET28a-*nfxB* plasmid was transferred into *E*. *coli* BL21 (DE3) to yield a recombinant strain. For NfxB production, the *E. Coli* cells were sub-cultured overnight in LB medium to OD_600_ = 0.6 at 37 °C; then, the protein was induced with 1 mM IPTG at 16 °C for another 16 h. Afterward, cells were harvested via centrifugation and lysed in His buffer A [10 mM Tris-HCl, 500 mM NaCl, 10% glycerol, pH = 7.5] with a JNBIO homogenizer (JNBIO, Guangzhou, China). The resulting cell lysis supernatant was affinity-adsorbed using a Ni-nitrilotriacetic acid (NTA) column (GE Healthcare, Piscataway, NJ, USA). After sufficient washing with 10% buffer A/buffer B [His buffer B: 50 mM Tris-HCl, 500 mM NaCl, 500 mM imidazole, 10% glycerol, pH = 7.5], the NfxB proteins were eluted using a linear gradient His buffer B. The final protein was desalted with His Buffer C [50 mM Tris-HCl, 300 mM NaCl, 10% glycerol, pH 7.5] using a HiTrap Desalting column (GE Healthcare, Piscataway, NJ, USA). The resulting proteins were analyzed using SDS-PAGE and stored at −80 °C until use.

### 4.8. Electrophoretic Mobility Shift Assay (EMSA)

DNA probes were amplified from the *hcp1* or *mexC* promoter region with PCR using the corresponding primers listed in Table S2. Then, 0, 0.05, 0.1, or 0.15 μM NfxB proteins were mixed with 1.33 ng/μL of *hcp1* or *mexC* promoter probes in 15 μL of Tris buffer [10 mM Tris-HCl, 50 mM KCl, 5 mM MgCl_2_, 3 μg/mL ssDNA, pH = 7.0] at room temperature. After 10 min of incubation, 2 uL of DNA gel shift loading buffer was mixed with reaction samples, and 10 μL of each sample was loaded onto 8% native polyacrylamide gel, followed by electrophoresis in 0.5×TBE buffer at 90 v for 1.5 h. Finally, the gel was stained with SYBR Gold dye (TransGene, Beijing, China), and the DNA bands were visualized with a Tanon 5200 Image analysis system (Tanon Technologies, Shanghai, China).

### 4.9. Pyocyanin Production and Biofilm Formation Assay

Pyocyanin was extracted and measured as previously described [40]. Briefly, 3 mL of chloroform was added to 5 mL of bacterial culture supernatant. After sufficient vortex mixing and standing for 10 min, the chloroform supernatant was mixed with 1 mL of 0.2 M HCl. The mixture was centrifuged to remove the superstratum, the absorption of the substrate was measured at 520 nm, and the pyocyanin production/culture supernatant (μg/mL) was determined by multiplying the OD_520_ by 17.072.

Biofilm formation was measured as previously described [41]. Briefly, *P. aeruginosa* cells were sub-cultured overnight in LB medium to 1% at 25 °C for 20 h in borosilicate tubes. Cell cultures were removed with a pipette, and the biofilms were stained with crystal violet (0.1%). After washing with water, the biofilms were dissolved with 0.5 mL of 95% ethanol, and the absorbance was measured at 600 nm.

### 4.10. Antibiotic Susceptibility Assay

To detect the antibiotic susceptibility of the *P. aeruginosa* strains, overnight cultures were normalized and plated on solid LB agar. Paper filter disks (3 mm) with 10 μL of the indicated concentration of antibiotic solution were placed on the plates and incubated at 37 °C for 12 h. Then, the bacterial growth inhibition zones were imaged, and the diameter of the clear zone around each filter disk was measured. For minimum inhibitory concentration (MIC) detection, overnight bacterial cultures were normalized to an OD_600_ of 1.0 and diluted 100-fold with a fresh LB medium with a 2-fold gradient concentration of the antibiotic. After incubation at 37 °C for 18 h, the MIC of strain was determined according to the degree of liquid clarification and the dilution gradient.

### 4.11. Protein Secretion Assay

Secreted proteins were collected using methods described previously with minor modifications [32]. In brief, overnight *P. aeruginosa* cultures were diluted to 1% in fresh LB medium with continuous shaking at 37 °C until an OD_600_ of 1.0 was reached. Then, 500 μL of bacterial culture was pelleted and dissolved in 50 μL of 1×SDS loading buffer to obtain the cell samples. Next, 1 mL of cell supernatant was filtered to remove bacterial cells, and 100% trichloroacetic acid was added to the supernatant. After incubating on ice for 2 h, the samples were centrifuged and washed with ice-cold acetone three times. Finally, the pellets were dissolved in 30 μL of 1×SDS loading buffer to obtain the supernatant samples.

### 4.12. Bacterial Killing Assay

Bacterial killing assays were conducted as previously reported with minor modifications [21,42]. Briefly, overnight cultures of relevant *P. aeruginosa* strains and the E. coli competitor DH5α containing pMS402 (Tmp^r^) were diluted to OD_600_ = 2.0 or 0.4, respectively. Then, cells were mixed in a 5:1 ratio, and 10 μL of the cell mixture was spotted onto a 0.22 μm sterile filter paper on solid LB-LS medium. After 12 h of incubation at 37 °C, the bacteria were recovered and diluted to a suitable concentration with fresh LB, and the diluent was spread on LB plates containing trimethoprim. After culturing these plates overnight at 37 °C to obtain the CFUs, the recovered *E. Coli* cells were counted to analyze the killing ability of *P. aeruginosa*.

## Data Availability

Data are contained within the article and Supplementary Materials.

## Supplementary Materials

The following supporting information can be downloaded at: https://www.mdpi.com/article/10.3390/ijms26041472/s1. Reference [43] is cited in Supplementary Materials.
