# Supplementary material for: Regulation of the H1 Type VI Secretion System by the Transcriptional Regulator NfxB in Pseudomonas aeruginosa
Source: Int J Mol Sci. 2025 Feb 10;26(4):1472. doi: 10.3390/ijms26041472 (PMC11855083; doi:10.3390/ijms26041472)
Supplement: Supplementary file 1 [file ijms-26-01472-s001.zip › ijms-3399309-supplementary.pdf]

Table S1. Bacterial strains and plasmids used in this study.

Strain or plasmid	Relevant characteristics	Source
E. coli		
DH5α	Host for vector construction	Stratagene
BL21(DE3)	Host for expression vector pET28a	Invitrogen
P. aeruginosa		
PAO1	Wild type *Pseudomonas aeruginosa* PAO1	Lab collection
Δ*nfxB*	*nfxB* deletion mutant of PAO1	This study
Δ*nfxB*Δ*clpV1*	*nfxB/clpV1* double deletion mutant of PAO1	This study
Δ*nfxB*Δ*mexC*	*nfxB/mexC* double deletion mutant of PAO1	This study
Δ*nfxB*::*nfxB*	*nfxB* mutant complemented strain of PAO1	This study
**Plasmids**		
pEX18Ap	Gene replacement vector with pUC18 MCS; Ap^r	[39]
pEX18Tc	Gene replacement vector with pUC18 MCS; Tc^r	[39]
pEX18Tc-*nfxB*	*nfxB* deletion plasmid, pEX18Tc with upstream and downstream regions of *nfxB*	This study
pEX18Ap-*mexC*	*mexC* deletion plasmid, pEX18Ap with upstream and downstream regions of *mexC*	This study
pEX18Ap-*clpV1*	*clpV1* deletion plasmid, pEX18Ap with upstream and downstream regions of *clpV1*	[33]
pMS402	Expression reporter plasmid carrying the promoterless *luxCDABE* gene; Kan^r, Tmp^r	This lab
pKD-*fha1*	pMS402 containing the *fha1* promoter driving *luxCDABE* reporter gene	This study
pKD-*tssA1*	pMS402 containing the *tssA1* promoter driving *luxCDABE* reporter gene	This study
pKD-*hcp1*	pMS402 containing the *hcp1* promoter driving *luxCDABE* reporter gene	[43]
pKD-*tagJ1*	pMS402 containing the *tagJ1* promoter driving *luxCDABE* reporter gene	This study
pET28a	Expression vector with N-terminal hexahistidine affinity tag, Km^r	Invitrogen
pET28a-*nfxB*	Protein expression construct, the entire gene of *nfxB* cloned into pET28a vector	This study
mini-CTX-*lacZ*-Flag	Integration plasmid, mini-CTX-*lacZ* containing the 3x *flag* sequence, Tc^r	Lab collection
mini-CTX-*hcp1*-Flag	mini-CTX-*lacZ* containing the entire *hcp1* gene and the 3x *flag* sequence	This study

mini-CTX-*tse1*-Flag	mini-CTX-*lacZ* containing the entire *tse1* gene and the 3x *flag* sequence	This Study
mini-CTX-*nfxB*	mini-CTX-*lacZ* containing the entire *nfxB* gene sequence	This Study
mini-CTX-*nfxB*-*tse1*-Flag	mini-CTX-*lacZ* containing the entire *nfxB* gene with *tse1* gene and the 3x *flag* sequence	This Study
mini-CTX-*nfxB*-*hcp1*-Flag	mini-CTX-*lacZ* containing the entire *nfxB* gene with *hcp1* gene and the 3x *flag* sequence	This Study

Table S2. Primers used in this study.

Primer	Sequence (5'→3') [a]	Application
biotin-p*hcp1*-S	TCCAAGCTGCCTTCGGCCA	DNA pull down
biotin-p*hcp1*-A	GCCGATCTTGATGAACATATCAACA	
biotin-*hcp1*-S	ATGGCTGTTGATATGTTCATCAAGA	
biotin-*hcp1*-A	TCAGGCCTGCACGTTCTGGCGGATG	
pEX-*nfxB*-Up-S	TTTggatccGTCGGGGTTGGCGAACTT	Constructing *nfxB* deletion mutant
pEX-*nfxB*-Up-A	GGGctcgagGTCGACGATAGCGACTGC	
pEX-*nfxB*-Down-S	ATTctcgagTCCGCGCATACCCTGGAG	
pEX-*nfxB*-Down-A	ATTaagcttCGATTCCCGGCAGCAGGC	
pEX-*mexC*-Up-S	CCCaagcttAACAGCCAGAAGCCACTGTCG	Constructing *mexC* deletion mutant
pEX-*mexC*-Up-A	CCGctcgagGGCCATAGCCAACGCCCC	
pEX-*mexC*-Down-S	CCGctcgagGATCGTGCCGAAGCCGGA	
pEX-*mexC*-Down-A	CGCggatccGTAGATCACCCCCACCGAACC	
pKD-*fha1*-S	ATTctcgagGGCAGCCAGCAAAACGGGTA	Constructing *lux* reporter
pKD-*fha1*-A	ATTggatccGTAGCTGGTGATGGTCAATCGCA	
pKD-*tssA1*-S	ATTctcgagGTAGCTGGTGATGGTCAATCGC	
pKD-*tssA1*-A	ATTggatccGGCAGCCAGCAAAACGGGT	
pKD-*tagJ1*-S	ATTctcgagTGGAACATCCGCCAGAACGT	
pKD-*tagJ1*-A	ATTggatccGCGCCCGGATGCGAATGA	
pET-*nfxB*-S	CCGgaattcATGCGCACAATCAGAAAA	Protein expression
pET-*nfxB*-A	CCCaagcttTCAGGAGCGAGCCGGATT	
p*hcp1*-S	TCCAAGCTGCCTTCGGCCA	EMSA
p*hcp1*-A	GCCGATCTTGATGAACATATCAACA	
p*hcp1*(NfxB)-A	ATCTTTCCTCCCGTGTGGCTCG	
p*mexC*-S	ATCAGGGTCATCGATGGGTCCCG	
p*mexC*-A	ACGCAAATCAGCCATGACACACC	
CTX-*hcp1*-Flag-S	TTctcgagTCCAAGCTGCCTTCGGCCA	Western blot
CTX-*hcp1*-Flag-A	CGaagcttGGCCTGCACGTTCTGGCGG	
CTX-*tse1*-Flag-1-S	CTCggtaccGAACCACTCCTCACTCGGCGA	
CTX-*tse1*-Flag-1-A	ATCGAGACTGTCCATGGGGCGGGTTCTCCG	
CTX-*tse1*-Flag-2-S	CGGAGAACCCGCCCCATGGACAGTCTCGAT	
CTX-*tse1*-Flag-2-A	GACaagcttACTGGCCCTGGGCAGGCTGCA	
CTX-*nfxB*-S	CGGggtaccGACACACCCGACCGTTGATTG	Protein cloning
CTX-*nfxB*-A	CGGggtaccTCAGGAGCGAGCCGGATT	

[a] Restriction sites displayed in lowercase.

Protein	Description	Da	#
OprG	Outer membrane protein OprG precursor	25178	138
NfxB	Transcriptional regulator	21178	129
PA3309	Hypothetical protein	16543	86
Lrp	leucine-responsive regulatory protein	18970	36

Figure S1. List of the mass spectrometry results for proteins uniquely identified in the hcp1 promoter DNA fragment binding sample, with their mass and the number of identified peptides.

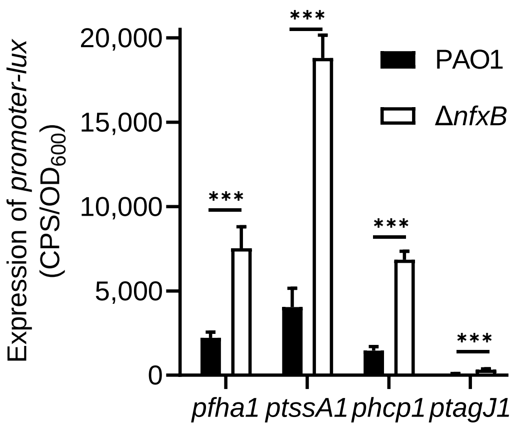

Figure S2. The indicated promoter activity of H1-T6SS were measured in wild-type PAO1 and Δ*nfxB* mutant strains. The indicated bacteria were cultured in LB medium at 37°C for 12 hours. The expression of *fha1-lux, tssA1-lux, hcp1-lux, tag1-lux* and optical density (600 nm) of each sample were obtained at 37°C for 12 hours. Data shown are the average of three independent experiments; error bars indicate SD from three independent experiments. Statistical significance was calculated using one-way ANOVA Dunnett's multiple comparison test, *** $p < 0.001$.

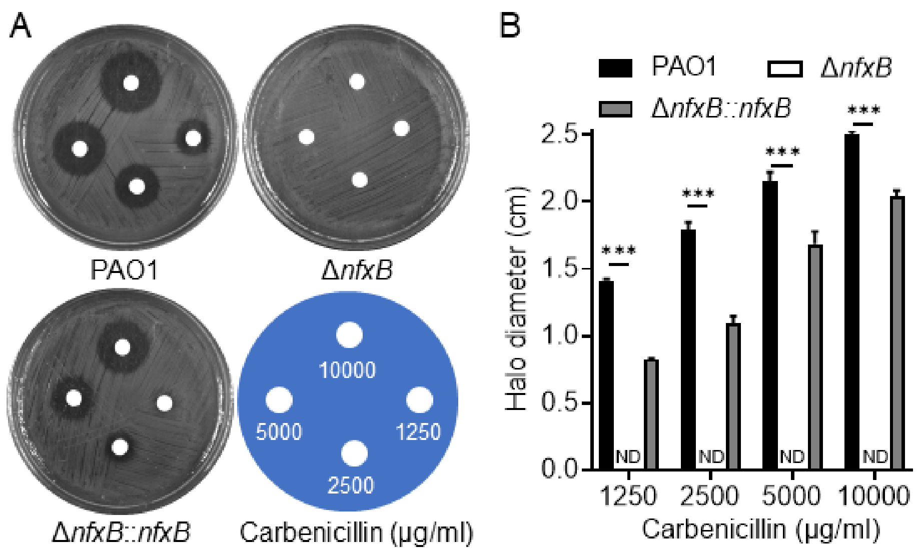

Figure S3. Deletion of *nfxB* increases carbenicillin resistance susceptibility of *P. aeruginosa*. Culture of the indicates wild-type PAO1, Δ*nfxB* mutant and the complemented strains were plated on LB solid medium. Then 10 μl indicates concentration of carbenicillin was spotted onto a paper disk. Plates were incubated at 37°C for 24 hours, and the photograph of clearing resistant zone were shown (A) and inhibition halo diameter were measured (B). (*A*) Similar results were obtained from three independent experiments, and the image shown are from one representative experiment. (*B*) Data shown are the average of three independent experiments; error bars indicate SD from three independent experiments. Statistical significance was calculated using one-way ANOVA Dunnett's multiple comparison test, *** $p < 0.001$.

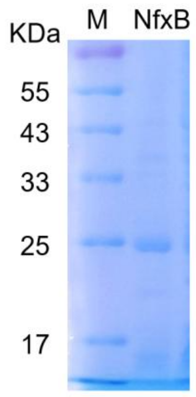

Figure S4. Gel electrophoretic analysis of purified NfxB by SDS-PAGE. SDS-PAGE gel was stained by coomassie Blue R-250. M: protein marker. NfxB: purified His_6-NfxB.
